# Tau different conformers; seeds ‐ how do the first ones arise?

**DOI:** 10.1002/alz.085276

**Published:** 2025-01-03

**Authors:** Sofia Lövestam, Scheres and Goedert

**Affiliations:** ^1^ MRC Laboratory of Molecular Biology, Cambridge United Kingdom

## Abstract

**Background:**

Intermediate species in the assembly of amyloid filaments are believed to play a central role in neurodegenerative diseases and may constitute important targets for therapeutic intervention.

**Method:**

We used time‐resolved cryogenic electron microscopy (cryo‐EM) to study the in vitro assembly of recombinant truncated tau into paired helical filaments of Alzheimer’s disease or into filaments of chronic traumatic encephalopathy.

**Result:**

We report the formation of a shared first intermediate amyloid filament, with an ordered core comprising residues 302‐316. At later time points, the first intermediate amyloid disappears and we observe many different intermediate amyloid filaments, with structures that depend on the reaction conditions. At the end of both assembly reactions, most intermediate amyloids disappear and filaments with the same ordered cores as those from human brains remain.

**Conclusion:**

Tau filaments assemble via polymorphic intermediates.

